# Comment on the Correlation between Complete Blood Count Parameters and Appendix Diameter for the Diagnosis of Acute Appendicitis

**DOI:** 10.3390/healthcare8040461

**Published:** 2020-11-05

**Authors:** Sami Akbulut, Tevfik Tolga Sahin

**Affiliations:** Department of Surgery and Liver Transplant Institute, Faculty of Medicine, Inonu University, 44280 Malatya, Turkey; tevfiktolgaa@gmail.com

## To the Editor:

We read the recent article published by Daldal and colleague with great interest [1]. The authors claimed that a complete blood count parameter evaluation with the clinical findings revealed that Neutrophil to Lymphocyte Ratio (NLR) is an important parameter that may help the diagnosis of acute appendicitis with an appendix diameter of >6 mm. We would like to share our opinion and criticisms about this study.

We found that a significant mistake was made in grouping the patients. This mistake will cause bias in the entire data of the article. It is widely accepted that lymphoid hyperplasia and fecaliths are the two most common causes of luminal obstruction leading to acute appendicitis. Therefore, in the present study, the classification of patients with lymphoid hyperplasia as a negative appendectomy is a serious mistake. The diagnosis of negative appendectomy is made by pathologic analysis of the appendix and shows that there are no findings that are consistent with inflammatory cell infiltration in the wall of appendix. For this reason, the presence of lymphoid hyperplasia in the patients does not rule out, histologically, the presence of acute appendicitis.

In Table 1, the authors have investigated the correlation between the diameter of the appendix and various parameters and have stated that some of the variables have shown significant correlations. However, if the Spearman’s rho correlation coefficients in Table 1 are analyzed in detail, even if the p values are less than 0.05, it is seen that there is no exact correlation or that the correlation is very weak. Therefore, this relationship between the variables is not prominent enough to be used in clinical practice.

The evaluation of the results in Table 2 shows that, if the appendiceal diameter is >6 mm, the risk of the development of acute appendicitis increases by 5.43-fold (95% CI = 2.2–13.1). Besides this, using the data provided in Table 2, the authors could have calculated the sensitivity (95%), specificity (21.7%), positive predictive value (88.4%), negative predictive value (41.7%), and accuracy (85%) for appendices with a diameter greater than 6mm in the prediction of acute appendicitis, which could have been very useful and formidable for the readers. Even though the authors have not mentioned these results, we believe that it is very important for readers to know these results. Similarly, the analysis of the scatter plots in Figure 1 shows that there is almost no correlation between NLR versus diameter and platelet to lymphocyte ratio (PLR) versus diameter.

In Table 5, the authors have performed a receiver operator characteristics (ROC) curve analysis to calculate t0he optimal cut-off values for leukocytes (WBC), neutrophils (N) and platelet distribution width (PDW) in patients with an appendiceal diameter over 6 mm. However, in the statistical analysis section, the authors have stated that they have performed a ROC curve analysis to determine the optimal cut-off value for the diagnosis of acute appendicitis. For this reason, the statement in Table 5 is wrong and the table should be re-organized according to the aims stated in the statistical analysis section. Furthermore, the authors state that the optimal cut-off values for the WBC, N, PDW, and NLR would be calculated, but the cut-off values have not been used in any of the c calculations that are expressed in the table. If the values are not going to be evaluated according to the cut-off values than there is no need for the ROC curve analysis.

The heading of Table 3 states that ‘‘Comparison of CBC parameters in groups divided by appendix diameter’’. However, in Table 3 the parameters are not distributed according to appendiceal diameter, but they are distributed according to the pathologic results of the appendectomy specimens, which is acute appendicitis versus lymphoid hyperplasia. The authors have stated that they have determined the diameter of the appendix using either CT or ultrasonography images. Using either of the two different imaging techniques causes heterogeneity regarding the diameters of the appendix vermiformis. Ultrasonography is operator-dependent and it requires certain expertise for the technique; on the other hand, CT images are more objective. For these reasons, including both techniques for the evaluation of the appendiceal diameter results in bias in the present study.

In the conclusion section, the authors have stated that ‘‘assessment of appendix diameter and complete blood count parameters can be used together to increase the diagnostic value of acute appendicitis’’, but this statement does not comply with the results that the authors have found, because the authors have found a very weak relationship with the parameters that have been investigated. For this reason, we cannot make any inferences from the results of the present study.

In our opinion, the authors’ approach should have been to determine the optimal cut-off values for the diameter of appendix vermiformis and laboratory parameters according to the histopathological diagnosis of acute appendicitis. Furthermore, after the determination of the optimal cut-off values, the factors that affect the prediction of acute appendicitis should have been evaluated.
